# Unusual case of severe arrhythmia developed after acute intoxication with tosylchloramide

**DOI:** 10.1186/2050-6511-14-8

**Published:** 2013-01-24

**Authors:** Vincenzo Lariccia, Alessandra Moraca, Marco Marini, Annamaria Assunta Nasti, Ilaria Battistoni, Salvatore Amoroso, Gian Piero Perna

**Affiliations:** 1 Department of “Biomedical Sciences and Public Health, University “Politecnica delle Marche”, Ancona, Italy; 2 Department of Cardiology, Azienda Ospedaliero-Universitaria “Ospedali Riuniti di Ancona”, Ancona, Italy

**Keywords:** Tosylchloramide, Acute intoxication, Arrhythmia, Ventricular fibrillation, Ion channels

## Abstract

**Background:**

Drugs not commonly considered to be cardioactive agents may cause prolongation of the QT interval with resultant torsades de pointes and ventricular fibrillation. This form of drug toxicity often causes cardiac arrest or sudden death.

**Case presentation:**

After accidental ingestion of tosylchloramide a caucasian 77-year-old woman, with a family history of cardiovascular disease and hypertension, was admitted to the intensive care unit following episodes of torsades de pointes with a prolonged QT/QTc interval (640/542 ms). The patient received an implantable cardioverter-defibrillator, was discharged from the hospital with normal QT/QTc interval and did not experience additional ventricular arrhythmias during one year of follow-up.

**Conclusion:**

This is the first report concerning an unusual case of torsades de pointes after accidental intoxication by ingestion of tosylchloramide. The pronounced impact of the oxidyzing agent tosylchloramide on the activity of some of the ion channels regulating the QT interval was identified as a probable cause of the arrhythmia.

## Background

Because of its high activity against fungi and bacteria, tosylchloramide is a widely used disinfectant agent for common applications such household cleaning and swimming pool disinfection. Many case reports describing tosylchloramide intoxication have been already published in the past showing that the chronic exposure to this compound may cause hypersensitivity reactions, such as asthma [1,2], conjunctivitis [3], whereas toxic pneumonitis [4], cardiovascular collapse and myocardial damage may occur in acutely intoxicated patients [5]. Here we report the first case of a severe arrhythmia developed in the context of acute oral intoxication with tosylchloramide.

## Case presentation

**Figure 1:**
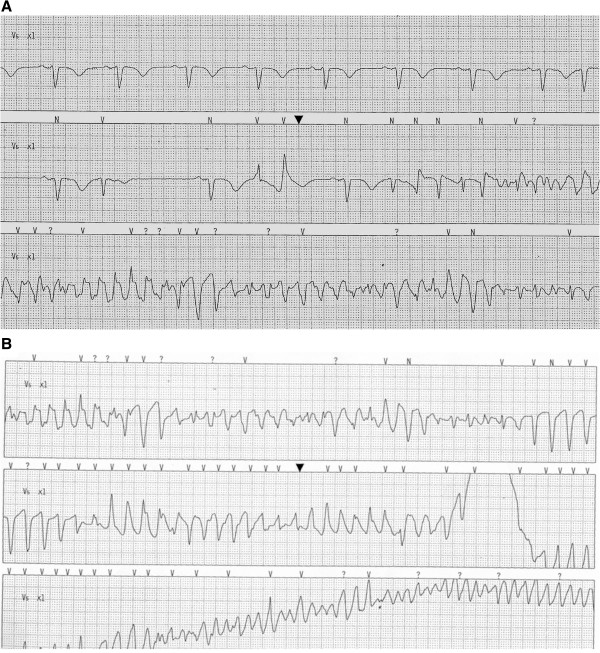
**TDP with fast degeneration into Ventricular Fibrillation.** The ECG-strip pre-TDP (Figure1A) shows a QT interval of 640 msec (QTc is 542 msec).

A 77-year-old woman presenting shoulder girdle pain was admitted to our hospital with suspected coronary syndrome. She had a history of hypertension; treated since 5 years with Perindopril (5 mg once daily) and a family history of cardiovascular disease. Few hours after the admission at the Emergency Room (ER) the patient experienced a cardiac arrest due to a “Torsade de Pointes” (TdP) degenerated into ventricular fibrillation which required DC shock (200 J), as documented by electrocardiogram (ECG) (Figure 1). After specific questioning for drug intake, the patient revealed She had accidentally (non-intentionally) ingested an entire sachet of Euclorina (containing 2.5 g of tosylchloramide) between 5 and 6 hours before TdP.

**Figure 2:**
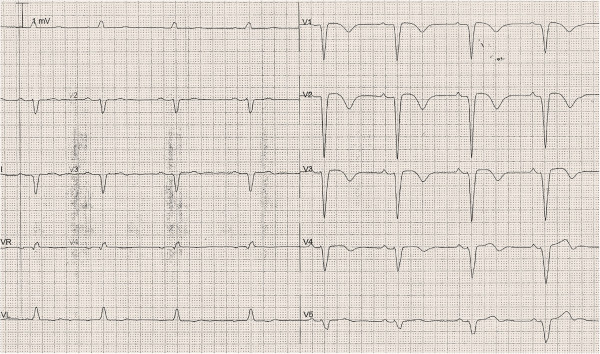
ECG at the admission to ICU shows a LBBB with a prolonged QT/QTc interval (640–542 msec).

Surface ECG on admission in ER showed sinus rhythm with pre-existing left bundle branch block (LBBB). Serum potassium was in slightly lower normal range (3.3 mEq/l, before 3.8 mEq/l), while other haematological parameters were in their respective reference intervals (data not shown). On admission to the cardiology Intensive Care Unit (ICU) the ECG disclosed sinus bradycardia (55 bpm) with significant alteration in the QT interval (QT/QTc = 640/543 msec; Figure 2) that was significantly prolonged over a 24-hour period. Thereafter the patient underwent an echocardiography that revealed concentric left ventricle hypertrophy with a mild reduction in global systolic function (LVEF = 50%) due to LBBB-induced dyssynergy of the interventricular anterior septum wall.

**Figure 3:**
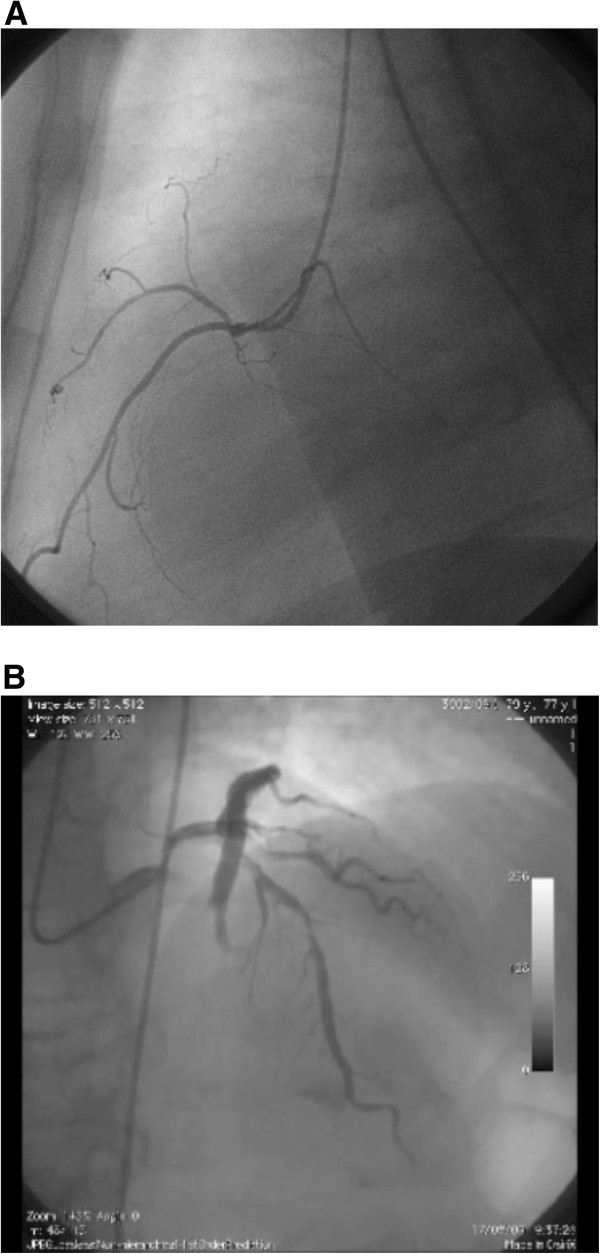
**Coronary Angiography.** No significant CAD is detected in right cororary artery. The middle left anterior descending coronary artery had a non-significant stenosis (< 50%), as confirmed by IVUS control.

The middle left anterior descending coronary artery (LAD) had a non-significant stenosis (< 50%) as revealed by coronary angiography and IVUS control (Figure 3). A not significant stenosis was detected in a non-dominant right coronary artery (Figure 3). According to the criteria defined in Thygesen et al. [6], myocardial infarction was excluded for the following reasons: a) no symptoms or electrocardiographic changes were detected; b) the peak CK-MB level was 8.1 ng/ml (the normal reference value in our laboratory is < 5 ng/ml) only after 6 h from DC shock, and normalized (3.2 ng/ml) within the following 6 h; c) the peak Troponin I level was 0.32 ng/ml (the normal reference value in our laboratory is < 0.08 ng/ml) 6 h after DC shock, and normalized (0.06 ng/ml) 6 h later. No signs of liver or kidney derangement were observed (data not shown).

**Figure 4:**
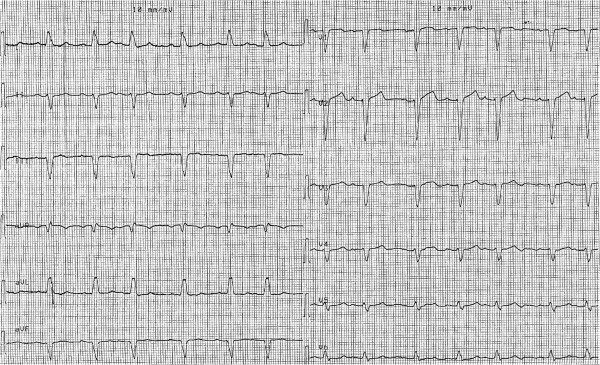
**ECG at hospital discharge.** QT interval measures 400 msec, the QTc interval 430 msec.

**Figure 5:**
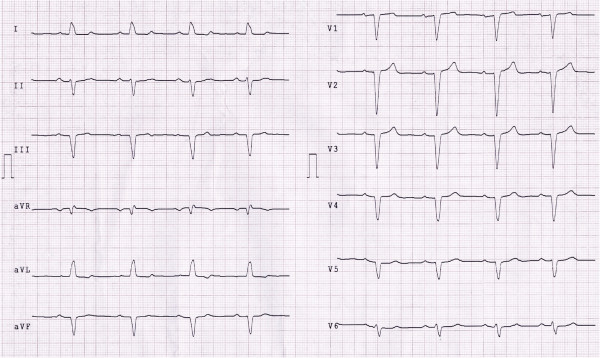
ECG at 1-year follow-up shows stable LBBB and normal QT/QTc interval (405/432 msec).

The patient was discharged 7 days after admission, following the placement of an implantable cardioverter-defibrillator (ICD); the ECG at discharge showed a LBBB with a normal QT interval (QT/QTc = 400/430 msec; Figure 4). After 12 and 24 months of follow-up She has been clinically stable, no shock detected at ICD registration and her QT interval was normal (Figure 5).

## Discussion

The occurrence of TdP in our patient after tosylchloramide ingestion can be explained considering that this compound acts as a strong oxidant of methionines and cysteines residues in proteins [7]. The activity of some of the ion channels regulating the duration of the QT interval is, indeed, strongly influenced by oxydation of critical methionine residues in channel proteins. In particular, voltage-dependent inactivation of Na_V_ channels is significantly slowed down when methionine residues located in the so called IMF domain, which is responsible for voltage-dependent channel inactivation, are oxydized [8]. Consistently, tosylchloramide, which has a strong preference in oxydizing methionine residues [9], is one of the most powerful oxydants affecting Na_V_ channel inactivation [9,10] and it has been used as a pharmacological tool to abolish voltage-dependent inactivation in studies aiming to determine its contribution in the activity of specific cardiovascular drugs [11]. Tosylchloramide-induced slowing of Na_V_ channel inactivation is a quite general phenomenon being observed in the brain, muscle and, importantly, cardiac isoform of these channels [8]. Oxydant-induced impairment of Na_V_ voltage-dependent inactivation may *per se* explain the appearance of TdP in our patient intoxicated with tosylchloramide since it causes a marked increase in persistent I_Na_ (I_Na_P ) [12,13], the inactivation-resistant Na^+^ current which persists in the presence of prolonged membrane depolarization [14]. An increase in I_Na_P is, indeed, a well documented mechanism of QT prolongation and arrhythmogenesis and a potentailly relevant target for treatment and prevention of arrhythmias [15,16]. In addition, an increase in I_Na_P is considered responsible for arrhythmogenesis in patients affected with the LQT3 syndrome which bear specific mutations in the Na_V_1.5 channel gene [17-19]. Confirming the involvement of I_Na_P in oxydant-induced arrhythmogenesis, the I_Na_P blocker ranolazine was effective in preventing QT prolongation and early afterdepolarzations induced by the strong oxydant agent H_2_O_2_ in cultured guinea pig cardiomyocytes [13]. Therefore, it is tempting to speculate that, in our patient, tosylchloramide exposure recapitulated the pathophysiological mechanism of cardiac arrhythmia in LQT3 patients.

hERG is another oxydation-sensitive ion channel that could have been involved in the genesis of TdP in our patient. hERG is the main K^+^ current responsible for rapid repolarization of cardiac myocytes in phase III of cardiac action potential [20] and its loss of function is one of the best characterized mechanisms of drug-induced or congenital LQT syndrome [21]. Specifically, mutations causing either loss of function or alterations in trafficking of hERG channels are responsible for the LQT2 syndrome [22,23] whereas mutations in MiRP1, an accessory subunit that coassembles with hERG, have been found in LQT6 patients [24]. Intriguingly, by oxydizing critical methionine residues, tosylchloramide causes an almost complete loss of hERG channel activity *in vitro*[25] thus reproducing the effect of drugs or mutations known to cause TdP. Therefore, it is likely that hERG channel blockade could have played a role in the appearance of TdP in our patient.

Finally, it is worth to remind that several oxydants, including tosylchloramide, may also increase the activity of L-type voltage-gated Ca^2+^ channels (VGCC), even though, in this case, the specific inolvement of methionine residues has not been demonstrated [26,27]. By increasing Ca^2+^ influx through L-type VGCC, tosylchloramide is expected to prolong the plateau phase of cardiac action potential thus delaying cardiomyocyte repolarization and promoting the appearance of TdP. Intriguingly, an increase in L-type VGCC activity represents the mechanistic base of arrhythmias in LQT8 patients [28-30] bearing the Timothy syndrome mutations which cause an impairment in voltage-dependent Ca_V_1.2 channel inactivation [31].

## Conclusion

Oxidative stress has been proposed as one of the upstream events provoking clinical relevant arrhythmic responses [32] and several drugs used in therapy exert antiarrhythmic effects in part via their antioxidative property [33,34]. Here we suggest that severe arrhythmia may occur in the form of TdP after massive exposure to the oxidizing agent tosylchloramide. In fact, tosylchloramide has a pronounced impact on the activity of some of the ion channels regulating the QT interval. Since our patient exhibited no evidence of QT interval alteration after 12 and 24 months of follow-up, this strongly suggests a causal role of tosylchloramide intoxication for the ECG abnormalities occurred during observation in the ICU. Therefore, a strict electrocardiographic monitoring is advised in patients intoxicated with this compound.

### Consent

Written informed consent was obtained from the patient prior to publication of this case report and any accompanying images. A copy of the written consent is available for review by the Series Editor of this journal.

## Competing interest

The authors declare that they have no competing interest.

## Authors’ contributions

AM identified and managed the case; GPP and SA analyzed the data, conceived of the study and helped to draft the manuscript; VL, MM, AAN and IB performed the literature search and wrote the article. All authors read and approved the final manuscript.

## Authors’ information

Salvatore Amoroso and Gian Piero Perna equally contributed as senior authors.

## Pre-publication history

The pre-publication history for this paper can be accessed here:

http://www.biomedcentral.com/2050-6511/14/8/prepub
